# Development and Performance Evaluation of Enhanced Piezo-Electric Sensor Cum Energy Harvester Based on Flexural Strain Amplification in Real-Life Field Conditions

**DOI:** 10.3390/s25041063

**Published:** 2025-02-11

**Authors:** Sreenitya Singamsetty, Naveet Kaur, Suresh Bhalla

**Affiliations:** 1 Department of Civil Engineering, Indian Institute of Technology (IIT) Delhi, Hauz Khas, New Delhi 110016, India; sreenitya.singamsetty@civil.iitd.ac.in; 2 Bridge Engineering and Structures Division, CSIR-Central Road Research Institute (CRRI), New Delhi 110025, India; nkaur.crri@nic.in

**Keywords:** strain-amplifying sensors, structural health monitoring, energy harvesting, piezoelectric sensors, piezoelectric energy harvesting, bridge structures

## Abstract

Driven by technological advancements and accelerated infrastructure development, an increase in the need to monitor the performance of prominent structures such as bridges, metro-corridors, and sea-link bridges is being advocated by experts to predict and minimize any hazards resulting from the degradation of the structures over time. However, accessing and replacing the batteries becomes problematic and expensive when the sensors are instrumented in remote areas of the bridge structures, especially when the sensors are embedded. For these reasons, a strong case can be made for harvesting and storing ambient energy from the surroundings to drive the sensors for structural health monitoring (SHM). This study aims to introduce a new trapezoidal strain-amplifying sensor/energy harvester (TSAH) for civil engineering structures that uses flexural strain amplification to enhance energy harvesting from structural vibrations. TSAH also serves as a sensor for integrated energy harvesting and SHM. This article examines the influence of the geometric properties of TSAH on strain amplification via numerical investigations under a specific set of external loads. Based on numerical studies, the sensors are bonded to the trapezoidal strain-amplifying plate to develop and assess the TSAH. Experimental investigations were carried out first in the laboratory to evaluate the effectiveness of the TSAH over the directly bonded (DB) sensors with two different types of piezo-transducers for energy harvesting. The host structure was exposed to impact and shaker vibrations for the laboratory research. For the various scenarios taken into consideration in the study, the typical amplification factor for peak voltage is determined to be between 1.45 and 3.75, while for the power, it is between 1.09 and 6.08. Further, for field verification, the TSAH configuration was evaluated on a real-life bridge structure, viz the Chipiyana rail over-bridge (ROB), Asia’s heaviest steel ROB located on the Delhi–Meerut expressway. The field experiments also establish the superior performance of TSAH, with an amplification factor ranging from 1.75 to 3.75 for peak voltage and 3.75 to 5.53 for peak power. As compared to the previously proposed curved configuration in the literature, the TSAH configuration is suitable for brittle sensors as well. Its ability to be permanently bonded by epoxy/welding, or temporarily using magnets, bolts, or clamps, offers it versatility over other surface bonded/embedded configurations. As a result of this, it imparts reusability in case of any damage, which promotes the goal of sustainability.

## 1. Introduction

Energy harvesting is the process of capturing and converting any small amount of ambient energy from the surroundings into electrical or any other usable power. The ambient energy may be available in different forms such as vibrations, solar, and wind. In the civil engineering context, converting the energy from structural vibrations into useful electrical power to be used by sensors to monitor the health of the same structure has immense potential [1] for structures such as buildings, roads, rail bridges, flyovers, elevated metro corridors, and tunnels. The electrical energy harvested from the ambient vibrations can be stored for future use or may be used by the sensor in real-time health monitoring of the structures, using piezoelectric materials, such as lead zirconate titanate (PZT) and macro-fiber composite (MFC) patches. With the recent advances in wireless and micro-electro-mechanical systems (MEMS) technology, sensors can be instrumented at remote locations of the structures. For example, wireless sensors can be placed in remote locations, such as structural sensors placed on a bridge or global positioning service (GPS) tracking devices placed on animals in the wild. For wireless sensors, batteries are the source of a power supply with a finite life span. Hence, when the battery is exhausted, it is troublesome to replace. Detaching the sensor from the remote locations simply to replace the battery and re-instrumentation can become a very expensive task [2]. In civil structures, where embedded sensors are also used, battery replacement is almost impossible. In such cases, the energy harvested from the ambient vibrations, such as wind and structural vibrations, using PZT materials can be used to charge the battery and provide a continuous power supply to the sensors. Harvesting energy from ambient vibrations using the piezoelectric effect by converting mechanical energy to electrical energy is called piezoelectric energy harvesting (PEH) and is recognized as an emerging technique [3]. When compared to other modes of energy harvesting, PEH has gained prominence due to its higher power density [4].

Some prominent PEH-related research based on wind vibrations is described in this section. [5] evaluated experimentally the power generated using instantaneous wind and the intercepting area of the wind turbine. Ref. [6] explored energy harvesting from the vibrations caused by the flowing wind. A portable impact-type wind energy harvester was developed by [7], where a maximum power of 1.61 µW was measured using a 100 kΩ resistor by bonding MEMS-based harvesters on the free end of the cantilever. Refs. [8,9] harvested power of 1.19–3.8 mW from the wind vibrations from windmill-type energy harvesters. In these, the excitation was only in the axial direction of the piezoelectric material. Ref. [10] presented various wind energy harvesters developed by several researchers in their review paper [11,12,13,14,15,16,17,18,19,20], whose output power ranged from 363 µW to 8.5 mW. However, Ref. [21], in their paper, presented the power generated by the wind vibrations considering the torsional effect as well. The effect of the twisting, shape, and perforation of the cantilevers in power generation when subjected to wind was studied using a lab-sized wind tunnel, resulting in a power yield of about 7 µW. The main limitation of wind-based PEH is the compulsory requirement of a large secondary structure, which has restricted the widespread application of this technology.

Apart from the wind-based PEH covered above, in the last two decades, there has been rigorous research carried out in the field of PEH to scavenge the direct power generated from structural vibrations. Ref. [22] investigated piezoelectric materials as a potential power source for low-level vibrations. Ref. [23], through their theoretical analysis, validated and optimized a two-layer cantilever piezoelectric power generator. Ref. [24] investigated three different types of piezo-transducers experimentally for harvesting the power generated by the ambient vibrations. The study compared the energy harvesting capacity of the monolithic PZT, bimorph, and macro-fiber composite (MFC) by charging a battery. Ref. [25] investigated power scavenging from low-level structural vibrations and succeeded by developing a small piezoelectric cantilever. Also, they optimized the design configuration to increase the power capacity. Ref. [26] developed a system that could generate power of 3.98 mW using an array of power generators made of piezoelectric cantilevers. Ref. [27] came up with a solution for low-frequency vibrations by proposing a silicon-proof mass on the PZT cantilever, which could generate an average power of 0.32 W and a power density of 416 W/cm^3^. Ref. [28] proposed a system that can be used for vibration control as well as energy harvesting. The above vibration-based PEH source also necessitated a secondary structure. Ref. [29] explored the possibility of energy harvesting from thin PZT patches in d_31_ mode without a secondary structure, and to use the same for structural health monitoring utilizing the harvested energy. It was found that the power in the micro-watt range could be harnessed in the idle time of the sensor, and the same could be utilized during SHM. The effects of losses such as mechanical damping, electrical damping, and shear lag were also studied in detail. They could generate 1.417 µW power against a peak displacement of 0.289 mm. In the same way, a concrete vibration sensor (CVS) is a ready-to-use PZT composite sensor for RC structures, and was initially developed as an SHM device by [30] to withstand the harsh conditions experienced during the construction. Unlike the surface-bonded PZT sensors, a CVS can withstand harsh weather conditions. It can pick ambient vibrations and also the earthquake-induced low-frequency structural vibrations. Ref. [1] evaluated the CVS as an energy harvester to achieve both energy harvesting and structural health monitoring from the same PZT patch by embedding it in concrete. Ref. [31] investigated harvesting energy from structural vibrations due to earthquakes. However, vibrations from earthquakes are not suitable for sustained PEH. Ref. [32] presented an extensive study to harvest energy from ambient vibrations, present over a wide frequency range, using a bi-stable vibration energy harvester.

In recent years, there has been a surge in the use of wireless sensors operating on batteries. To drive these sensors, energy can be harvested from the same sensors that are instrumented for SHM. In this field, extensive research has been carried out by researchers to harness the energy from structural vibrations induced by the traffic movement on the bridge structures. Ref. [33] theoretically investigated a highway bridge model with a moving single-point load by a linear single degree of freedom (SDOF) model of PEH. The results obtained could be potentially used in real-life scenarios to determine the energy harvested during vehicular motion. Ref. [34] designed an inertial power harvester that can convert bridge vibrations to electric energy. In general, most bridge vibrations induced by traffic movement are non-periodic with low frequency and low acceleration. Duly addressing the mentioned three challenges of real-life structures, the inertial harvester could scavenge an average power of 0.5 to 0.75 µW when tested on a suspension bridge. Ref. [35] formulated analytical equations for the transient response of the PZT patch to vehicular excitation. Ref. [36] developed a piezoelectric cantilever bimorph-based PEH which could successfully capture the low-amplitude, low-frequency vibrations of the bridge due to vehicular movement. Ref. [37] studied the behavior of pavement by modeling it as an infinite element Euler–Bernoulli beam considering the moving load as a line load. Further, investigations were carried out to scavenge energy from the vibrations caused by moving vehicles on the pavement system with the use of piezoelectric harvesters. It was observed that when the velocity of the moving load reached the critical velocity, the power harvested was at an optimum. Ref. [38] conducted experiments by bonding a piezoelectric patch to a simply supported beam with a moving mass replicating the vehicular movement. Due to the mass movement, voltage was generated across the piezo patch as the beam deflected. The analytical results were validated with the experiments and it was observed that the velocity of the moving mass played a significant role in the output voltage generated. Ref. [39] presented an analytical expression for energy harvesting from a simply supported beam when subjected to moving harmonic loads, including numerical studies. Ref. [40] proposed an approach to harvest the energy from bridge vibrations caused by moving trains under different operational scenarios. Further, they quantified the energy harvested from a railway bridge from a high-speed line for a duration of three and half hours during the passage of twenty trains. Ref. [41] proposed a nonlinear magnet-based energy harvester to augment the power generated from the vibrations of moving vehicles on a bridge. Depending on the vehicular speed, the distance between the magnet was varied to harvest the energy. Ref. [42] experimentally investigated the behavior of an energy harvester with different cross-sections on a bluff body for hybrid excitation. The difficulties encountered in energy harvesting related to civil engineering constructions are thoroughly described by [43]. They also provide details about numerous aspects, including the harvester’s shape and dimensions, placement strategy, materials, traffic circumstances, and geometrical design of the harvester, that affect the power output for PEH applications. Ref. [44] modeled 3D-printed plectrum configurations for specific power outputs. Further, numerical methods were used to analyze these outputs. Ref. [45] modeled energy harvesters by regulating the natural frequencies to enhance the energy capture in COMSOL Multiphysics. Power consumption is a challenge for the electronic devices of wireless sensors. The rapid development of circuits with ever-lower power consumption (in the range of micro-watt) in the field of applied electronics has encouraged the possibility of harvesting power from ambient vibrations, which is addressed by enhanced energy harvesting [46].

A lot of research has been carried out in the field of energy harvesting to increase the energy harvesting density by modifying the shapes of the harvesters or secondary structures. Ref. [47] contributed to inventing a novel ring-shaped harvester fabricated using a thin film made of nitride. They concluded that due to the torsional motion of the harvester, there was an increase in the frequency band. Ref. [48] employed the Doppler frequency shift technique to correlate the mechanical and electrical properties of the PZT sensor/actuator. Refs. [49,50] developed an L-shaped piezoelectric energy harvester to increase the frequency bandwidth. Ref. [51] designed a configuration consisting of a piezoelectric plate sandwiched between two trapezoidal plates (resembling a cymbal) that could amplify the strains and hence generate higher voltage for the same static loads. However, performance was not evaluated under dynamic loads, and energy harvesting potential was not explored. Ref. [52] discussed in detail the impact, benefits, and drawbacks of several piezoelectric energy harvester forms for footwear. Also, the study showed that a cymbalical (similar to a trapezoid) structure of the transducer amplifies the strain, thereby leading to increased power generation. Ref. [53] developed a concrete vibration energy harvester (CVEH) based on the curved configuration of the PZT patch. Ref. [54] explored the option of the curved configuration of the piezo sensors. They observed that curved sensors were able to scavenge much more power when embedded in reinforced concrete structures as compared to a straight configuration. The extensive literature review laid out the basis for identifying particular research needs and finalizing the specific objectives.

### Research Gaps and Novelty

From the above-presented literature, it can be noted that there has been a lot of research conducted on embedded energy harvesters and those employing large and complicated secondary structures. However, there are certain practical issues and limitations associated with the embedded harvesters. They are non-replaceable in case of any faulty performance or damage to the transducers. Further, they cannot be reused if their location needs to be changed. In addition to the aforementioned limitations, the use of embedded sensors is not feasible for steel constructions. Further, most piezoelectric transducers are brittle, and cannot be bent for attachment to the curved shape of harvesters; similarly, large secondary structures for PEH pose challenges such as high cost, loss of free space, and risk of damage. This article is focused on addressing the above limitations by introducing a non-rectilinear configuration of a very small-sized secondary structure. The proposed configuration is an adaptation and improvement of the trapezoidal static force sensor designed by Ref. [51]. The applicability of his design was limited to static loads only, through either direct or impedance measurements. The present study, on the other hand, proposes a far simpler configuration suitable for integration with steel as well as concrete structures. Unlike, Wang’s sensor, the proposed trapezoidal strain amplification harvester cum sensor (TSAH) is suitable for dynamic strain sensing and piezoelectric energy harvesting from real-life structures. This article extends the trapezoidal configuration for PEH by numerical and experimental investigations under static and dynamic loads, respectively. Additionally, laboratory studies were extended to the field to evaluate the application of the proposed TSAH to real-life civil engineering structures. Application to real-life bridges was not attempted in the past and is very crucial to understanding the industrial viability of the proposed concept.

This article proposes TSAH for attaining a cost-effective solution that enhances the sensitivity of the standard sensors by simple geometrical modifications. Due to its ability to amplify strain and address the practical issues imposed by the usage of embedded energy harvesters, a trapezoid-shaped secondary structure has been chosen. The trapezoid harvesters have several specific benefits, and the TSAH sensors cum harvesters are suitable for both flexible and brittle-type transducers. They can be used for steel construction as well. Since they are primarily externally attached, they can be replaced in case of any faulty performance of the sensors. The TSAH allows for replacement and reuse, which furthers the goal of sustainability. In the case of civil engineering structures such as buildings and bridges, the primary challenge is to capture the voltage generated from the ambient vibrations, as they are of very low amplitude and frequency Ref. [34]. The TSAH addresses this practical problem by amplifying the strains with simple geometric modifications of the secondary structure, leading to increased voltage generation at a minimal cost.

The effect of the geometric properties of the proposed sensor cum harvester, which amplifies the strain, was first evaluated in numerical studies. The experimental studies were then conducted using three different types of PZT patches: macro-fiber composites (MFCs), disc-type PZT, and square-type PZT, under both lab and field conditions. The following sections cover the comparative assessment of TSAH compared to directly bonded PZT patches.

## 2. Numerical Investigations

**Figure 1:**
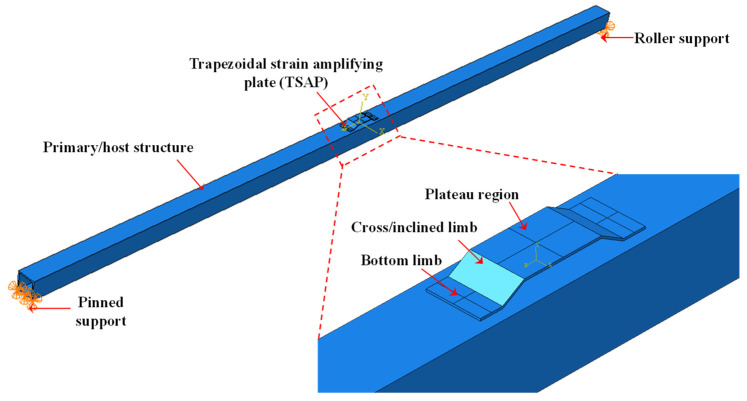
Geometric model of the beam assembly in ABAQUS^®^.

In this section, numerical investigations are covered to ascertain the strain amplification for given external loads by adopting the proposed TSAH. Basic numerical investigations were carried out in the finite element (FE) software, ABAQUS^®^, Version 6.14 [55] under the action of static load, to circumvent the limitations of the previously proposed curved-type energy harvester of Ref. [54], which cannot be instrumented on steel structures. The schematic diagram of the host structure and the trapezoid strain-amplifying plate (TSAP) is shown in Figure 1 and Table 1. It should be noted that sensors were not modeled in the numerical study as it is mainly focused on the effect of the geometric properties of the TSAP for strain amplification. All the dimensions mentioned in Table 1 are in mm. The dimensions of the TSAH were chosen to be the smallest possible such that it can accommodate different types of sensors such as MFC and PZT, which were considered for the investigations by not altering the physical properties of the primary structure such as mass and stiffness.

The details of the host structure were considered for a scaled lab-sized model of a foot over-bridge fabricated in the Smart Structures and Dynamics Lab (SSDL) at IIT Delhi [56].

The exterior longitudinal beams of the lab-sized model bridge act as the host structure for the current study. The numerical analysis of the longitudinal beam was carried out using finite element analysis of ABAQUS in the standard domain through its implicit integration technique.

**Figure 2:**
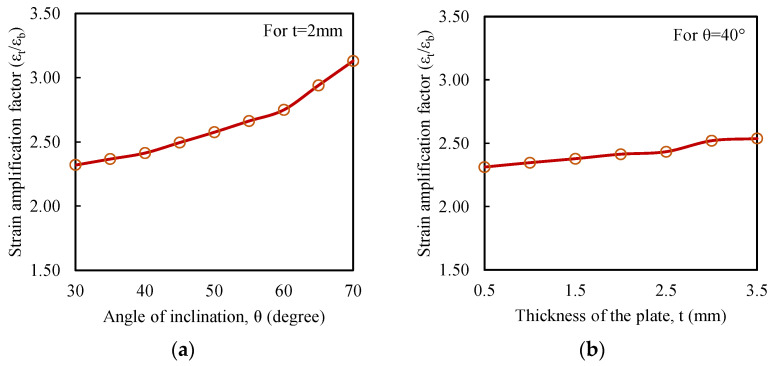
Variation in the strain amplification ratio with (**a**) angle of inclination and (**b**) thickness of the secondary TSAP.

Initially, the longitudinal beam and TSAP were modeled as separate parts in ABAQUS^®^ and later assembled ensuring a rigid connection for proper load transfer between the two parts. The longitudinal hollow square beam of length 3000 mm was modeled using 3D solid elements. The cross-section of the beam was first modeled in the XY-plane and then extruded in the Z-direction using the solid extrusion option available in ABAQUS^®^. Similarly, the longitudinal section was modeled first in the YZ-plane, and the solid extrusion was performed in the X-direction for the TSAP. The left edge of the host structure was restrained for translations in the X-, Y-, and Z-directions to simulate the pinned support, whereas the right edge was restrained for translations in the X- and Y-directions to simulate the roller support as shown in Figure 1. A uniformly distributed load of 1 N/mm^2^ was applied on the top surface in the XZ-plane in the negative Y-direction. The mesh size of finite elements was kept at 0.5 mm and the elements were modeled as a C3D8R, 8-node linear brick element for both the host and TSAP. Reduced integration with hourglass control was ensured for both entities. Initially, the angle of inclination (θ) of the cross limb in the TSAP was considered as 30° and the thickness of TSAP as 2 mm, as shown in Table 1. The strain amplification factor was arrived at by attaining the ratio of the strains on the plateau region (*ε*_t_) of the secondary structure to that on the host structure at a location below the TSAP (*ε*_b_) (see Table 1). Depending on the angle of inclination, the height *h* of the trapezoid plate was calculated by applying the *tan* rule as per the dimensions mentioned in Table 1. To ensure proper transfer of the loads from one entity to the other, the TSAP was bolted to the beam. The performance of the beam and TSAP assembly subjected to uniform pressure is interpreted in terms of the strain amplification factor (*ε*_t_/*ε*_b_). The finite element analyses were carried out by varying θ from 30° to 70° to determine the effect of the same on the strain amplification factor for a 2 mm thick plate. The variation in the strain amplification factor with the angle of inclination at an interval of 5° is presented in Figure 2a. The strain amplification factor was found to increase, on average, by 3.83% for every 5° increase in the angle θ. It is observed that the strain was amplified by 2.32 (min.) to 3.131 (max.) times when the angle of inclination was varied from 30° to 70°.

Similarly, the thickness of the trapezoid plate was varied from 0.5 mm to 2.5 mm at an interval of 0.5 mm and the factor was found to increase from 2.311 to 2.537. The effect of the thickness on the strain amplification is shown in Figure 2b for an angle of inclination of 40°. With the increase in the thickness of the TSAP, the strain amplification factor increased, but marginally. The increase in the strain is attributed to the shape of the TSAH and the increased distance of the plateau region of the TSAP from the neutral axis of the host structure.

The results of the numerical studies indicate that the trapezoid form increases the strains. Hence, several specimens of the TSAH were fabricated by bonding different types of PZT transducers to the secondary trapezoid structure for evaluation in laboratory and field applications.

## 3. Design and Fabrication of TSAH

**Figure 3:**
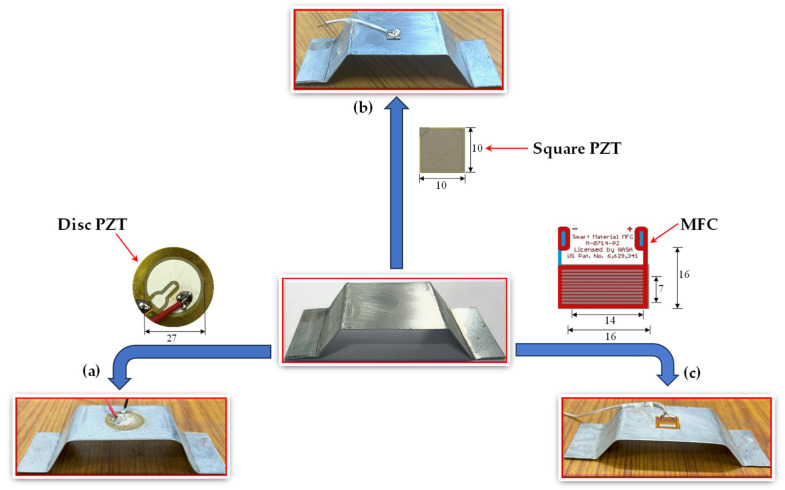
Sensors used for experimental laboratory and field investigations (**a**) TSAH-DISC, (**b**) TSAH-SQR, and (**c**) TSAH-MFC.

A rectangular mild steel plate of dimensions 162.5 × 50 × 0.5 mm was cut from a large plate using a cutting machine. As per the numerical study, the strain amplification factor increased marginally with the increase in the thickness; therefore, for the current experimental study, a 0.5 mm thick mild steel plate was selected considering the ease of bending. With the help of the bending machine, the cross limbs of the trapezoid were bent to achieve a moderate inclination of 40° with the horizontal. Since it is known from numerical studies that the amplification increases with an increase in angle, the angle of inclination was kept moderate for the experimental investigations. For the current research, four such specimens were fabricated to carry out both field and laboratory experiments; two were used for the laboratory experiments, and the other two for the field investigations. To conduct laboratory investigations, one TSAH named TSAH-DISC was bonded with a circular disc PZT patch (27 mm diameter and 10 mm width), manufactured by PRIELSYS [57]. The other, named TSAH-MFC, was bonded with macro-fiber composite (MFC), manufactured by Smart Materials Corporation [58], as shown in Figure 3. For the current study, MFC with the *d*_31_ variant labeled M-0714-P2 with dimensions (Figure 3c) was used with a thickness of 300 µm. For field investigations, one TSAH was bonded with a circular disc PZT patch, i.e., TSAH-DISC, and the other with a square PZT patch (10 × 10 × 0.3 mm) of grade PIC 151, manufactured by PI Ceramic [59], named TSAH-SQR.

## 4. Methodology for Laboratory Investigations

### 4.1. Framework of Experiments

**Figure 4:**
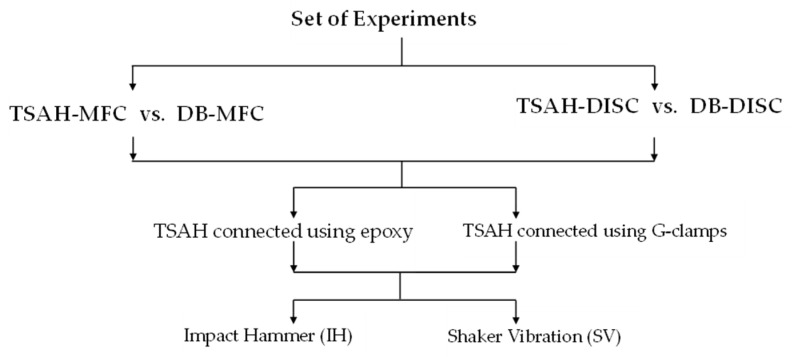
Flow chart of the experiments carried out for voltage and power measurement.

**Figure 5:**
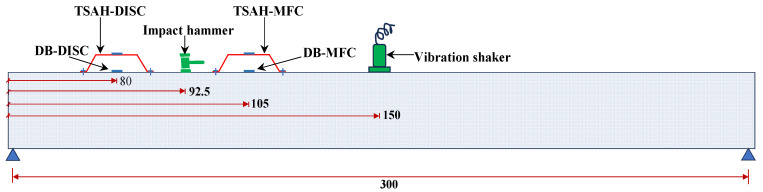
Schematic diagram of the host structure showing locations of PZT-based harvesters cum sensors and excitation points (all the dimensions mentioned are in cm).

A lab-size model of the Panchsheel foot over-bridge located near IIT Delhi, fabricated in Smart Structures and Dynamics Lab (SSDL), IIT Delhi, was used to perform the lab experiments. The structure is a steel-type foot over-bridge consisting of exterior and interior longitudinal beams, and cross-bracings [56]. The flow chart shown in Figure 4 summarizes the entire set of experiments conducted as part of lab benchmark studies. In general, the TSAH can be connected to the host structure by using G-clamps, bolts, welding, or bonding with epoxy. In this study, two connection methods were mainly evaluated (see Figure 4): (a) permanent bonding using epoxy adhesive and (b) detachable/reusable type connection using G-clamps. The experiments were carried out by subjecting the host structure to two different types of excitations: (i) impact hammer (IH) and (ii) shaker vibration (SV). The strain amplification potential using the TSAH sensors was evaluated for the different connection criteria under IH and SV excitations. A hammer was used to induce the impact vibrations. The TSAH was bonded to the exterior longitudinal beam such that the directly bonded (DB) transducer was exactly below it and the centroids of both the harvesters lay in one vertical line. The locations of all four PZT-type harvesters used in the experimental study are shown in Figure 5. The host structure was subjected to hammer excitation equidistant/midway from both the sensors, i.e., at 92.5 cm from the left support. A low-cost mechanical shaker developed at SSDL, IIT Delhi using two-stroke engines, was used to impose harmonic vibrations on the host structure [60]. This vibration shaker was positioned exactly at the center of the lab-sized model, i.e., 150 cm from the center of the support. The maximum force generated by this shaker, which operated at a frequency of 24 Hz, was 136 N. The vibration excitation positions of the hammer and shaker were chosen to be different to demonstrate that the strain amplification occurs regardless of the excitation location.

### 4.2. Impedance Matching Technique for Power Measurement

The impedance of the piezo-transducers is generally high, typically in the mega-ohm range. Due to the high mechanical impedance of the transducer, the current flowing (*i*) through the circuit is very small and is difficult to measure directly in an accurate manner. As per this technique, to minimize the losses and to achieve maximum power transfer, the impedance of the load resistors needs to be matched as best as possible with the impedance of the source impedance [61].

**Figure 6:**
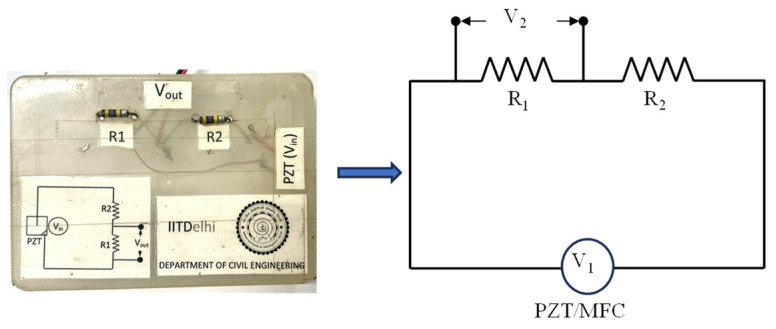
Schematic representation of power measuring circuit.

The load resistors, *R*_1_ and *R*_2_, of the circuit, as shown in Figure 6, were chosen based on the impedance matching technique. Hence the output voltage (*V*_2_) across the resistor, *R*_1_, was measured using a digital multimeter of high resolution to accurately determine the current flowing through the circuit and the power developed due to the excitations. The voltage generated across the transducer when the structure was subjected to excitations was fed as the input voltage (*V*_1_) to the circuit. The output voltage (*V*_2_) was measured across the resistor, *R*_1_. From the output voltage (*V*_2_), the current (*i*) and the power were calculated using

(1) $$i=\frac{V_{2}}{R_{1}}$$

(2) $$P=i^{2}(R_{1}+R_{2})$$

**Figure 7:**
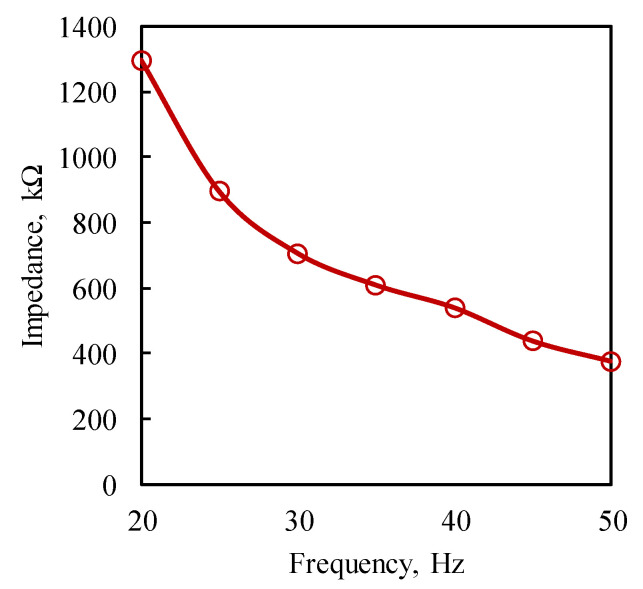
Variation in the impedance of MFC with the frequency.

It was observed that the impedance decreases as the frequency of vibration increases (see Figure 7). From the graph plotted, the interpolated value of the impedance at the excitation frequency of 24 Hz was determined as 974.594 kΩ, as the shaker induces vibration at this particular frequency.

## 5. Experimental Investigations for Lab Benchmark Study

### 5.1. Experimental Set-Up

**Figure 8:**
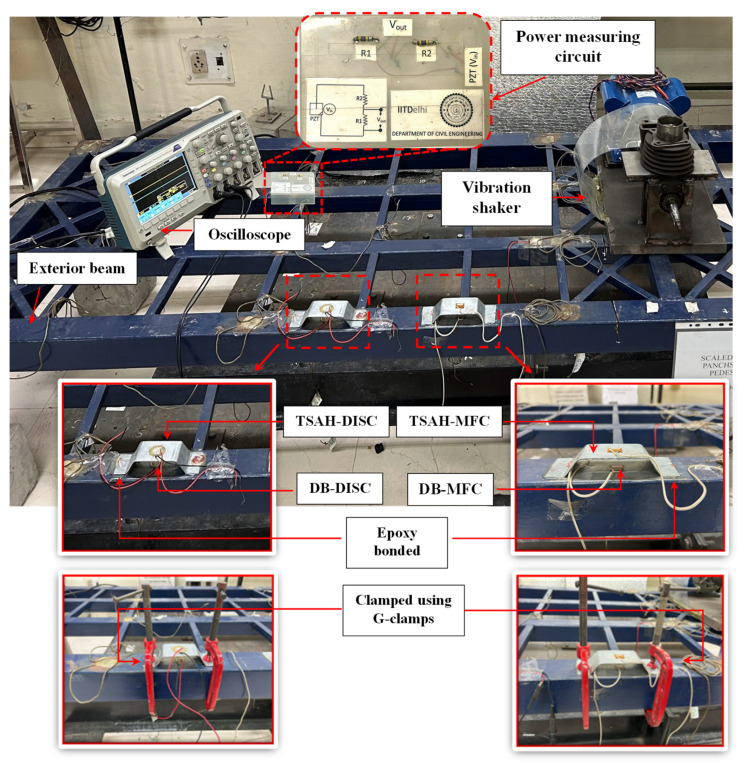
Experimental setup for the measurement of open circuit voltage and voltage across the power circuit.

All the PZT-based harvesters cum sensors considered in this study were bonded on the exterior longitudinal beams of the prototype bridge structure, as shown in Figure 8. The longitudinal beam of the lab-sized steel foot over-bridges, which is 3 m long (c/c), acts as a host structure. It was instrumented with TSAH and directly bonded (DB) PZT transducers to evaluate the relative energy harvesting potential of the proposed strain amplification approach. The experiments were conducted to determine the open circuit voltage of all four PZT-based transducers. The measurement was made using a TDS 2004B oscilloscope [62]. Further, the power generated across the PZT-based harvesters cum sensors was measured by connecting an auxiliary impedance-matched circuit, known as a power measuring circuit (see Figure 6).

### 5.2. Efficacy of TSAH Using Macro-Fiber Composite (MFC) Sensors as Potential Energy Harvesting Device

**Figure 9:**
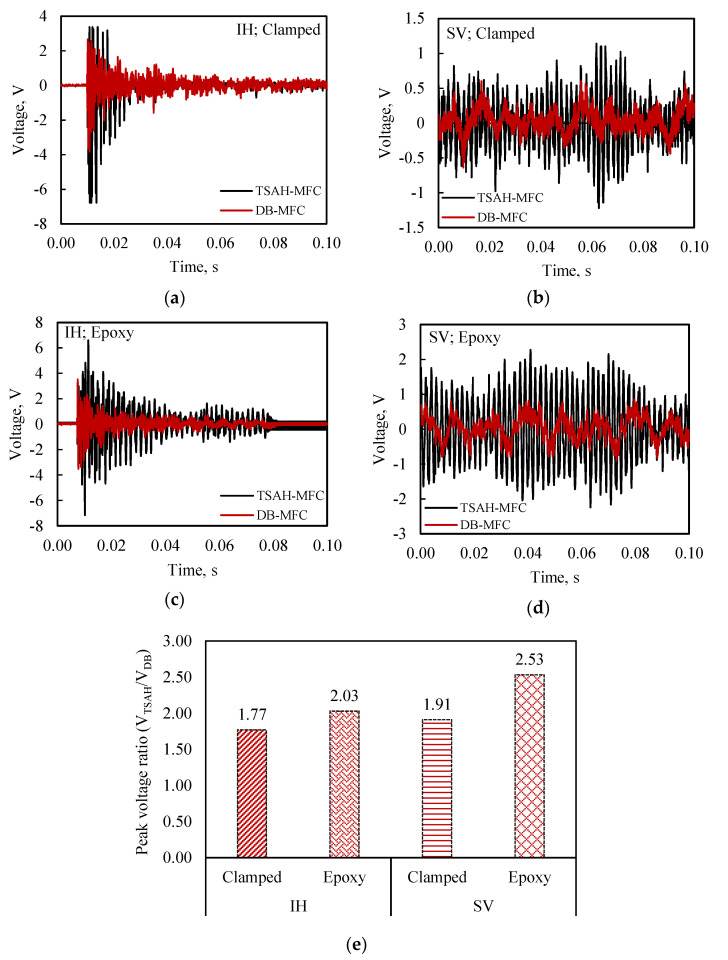
Variation in open circuit voltage with time for TSAH-MFC and DB-MFC, with the host structure subjected to (**a**) impact hammer (IH) vibrations (clamped), (**b**) shaker vibrations (SV) (clamped), (**c**) impact hammer (IH) vibrations (adhesively bonded), and (**d**) shaker vibrations (SV) (adhesively bonded); (**e**) peak voltage ratio (V_TSAH-MFC_/V_DB-MFC_) for all four conditions.

Initially, the open circuit voltage of the piezo-transducers and the power generated across an impedance-matched circuit were measured with the TSAH-MFC sensor connected to the host structure using G-clamps; and the DB-MFC sensor was directly bonded to the host structure (see Figure 8) for both IH and SV excitations. Subsequently, the G-clamps were removed and the TSAH-MFC was permanently bonded to the host structure using epoxy adhesive and the measurements were repeated; the results are presented in Figure 9. The above measurements were acquired for a duration of 0.1 s.

#### 5.2.1. Measurement of Open Circuit Voltage

The open circuit voltage of the TSAH-MFC and the DB-MFC was measured when the host structure was subjected to IH and SV excitations under clamp connection criteria using an oscilloscope. The voltage responses of the TSAH-MFC and the DB-MFC are shown in Figure 9a and Figure 9b for IH and SV vibrations, respectively. The experiments were repeated by removing the G-clamps and bonding the TSAH-MFC to the host structure using a two-part epoxy adhesive. Figure 9c and Figure 9d depict the voltage response when the TSAH-MFC was adhesively bonded to the host structure. The plotted graphs show that in every case, a greater voltage response across the TSAH was obtained compared to the directly bonded MFC patches. In the current study, the peak voltage ratio (V_TSAH-MFC_/V_DB-MFC_) was a metric used to quantify strain amplification.

Figure 9e summarizes the findings using the peak voltage ratio, where the peak voltage ratio for the above-mentioned cases ranges from 1.77 to 2.53. The shape and configuration of the secondary structure are thought to be responsible for voltage amplification and improved strain transfer.

#### 5.2.2. Power Measurements

**Figure 10:**
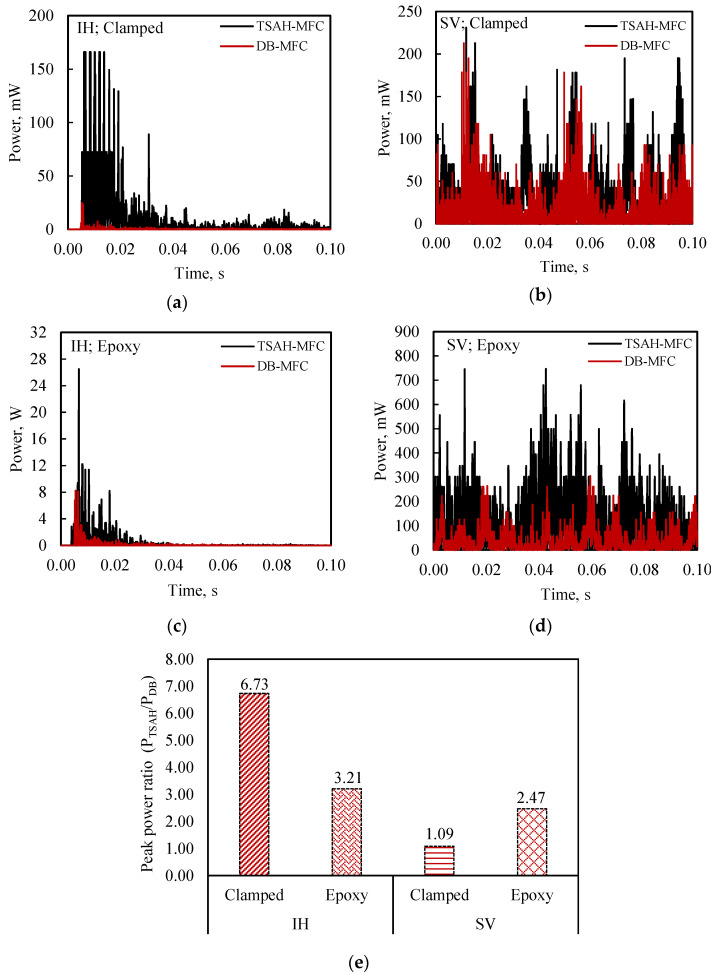
Comparison of power generated between DB-MFC and TSAH-MFC, with the host structure subjected to (**a**) impact hammer (IH) vibrations (clamped) and (**b**) shaker (SV) vibrations (clamped); (**c**) impact hammer (IH) vibrations (adhesively bonded) and (**d**) shaker (SV) vibrations (adhesively bonded); (**e**) peak power ratio (P_TSAH-MFC_/P_DB-MFC_) for all four conditions.

In this, the power measuring circuit (see Figure 6) was used to determine the power generated by the sensors under different vibrations. The output voltage, *V*_2_, of the power circuit was measured using an oscilloscope for an input voltage, *V*_1_, of the PZT transducers. The power generated was computed using Equation (2) for the various scenarios shown in the flow chart (see Figure 4). Figure 10a and Figure 10b depict the power generated by the TSAH-MFC and the DB-MFC, respectively, when the host structure was tested with IH and SV excitations under clamp conditions. Figure 10c and Figure 10d show the power generated for the adhesive bonding case under IH and SV excitations, for a duration of 0.1 s, respectively. The presented graphs demonstrate that, compared to the directly bonded MFC patches, a higher power was achieved across the TSAH.

The peak power ratio (P_TSAH-MFC_/P_DB-MFC_) was adopted as an indicator to quantify the power amplification. The peak power ratio for the above-mentioned cases is summarized in Figure 10e. It can be observed that the power ratio was substantially high for the TSAH-MFC when compared with the DB-MFC. From Figure 10e, it can be observed that the power generated across the circuit for the clamped TSAH-MFC is 6.5 (approx.) times the DB-MFC under IH vibrations. This may be attributed to additional fluttering of the TSAH on sudden hits due to the impact type of excitations. Unusually high values of the power ratio, observed in the case of clamped conditions, may be attributed to the variability accompanying the force transfer to the TSAH owing to the temporary nature of connections.

### 5.3. Feasibility of Off-the-Shelf PZT Transducers for Energy Harvesting

MFC is a highly top-notch specialized PZT sensor. In this study, experiments were also conducted to explore the performance of off-the-shelf disc-type PZT patches commercially available on merchandise sites like Amazon. Circular disc PZT patches procured from PRIELSYS (2024) were used and the experiments were repeated by replacing the MFC patches with disc PZT patches. The typical cost of the disc PZT was USD 2.39 in comparison to the approximate cost of USD 48.00 for MFC patches. The geometry of the disc-type PZT patches is shown in the earlier sections of this article (see Figure 3). The placement of the disc-type PZT sensors and the excitation location are shown in Figure 5. Initially, the experiments were conducted by attaching the TSAH-DISC to the host structure using G-clamps for both IH and SV excitations.

**Figure 11:**
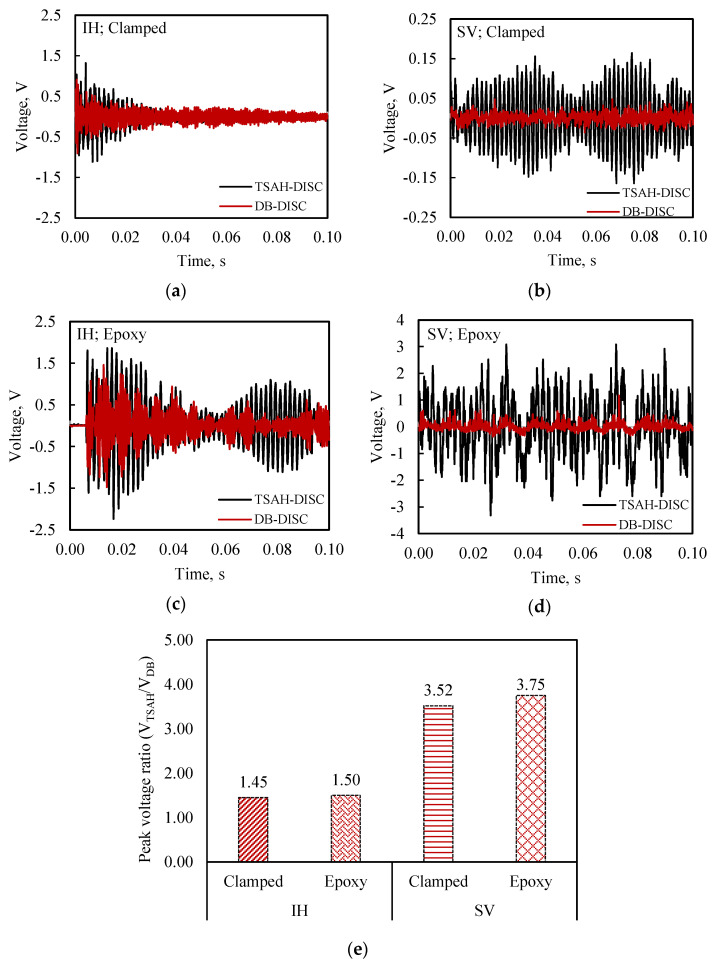
Variation in open circuit voltage with time for TSAH-DISC and DB-DISC, with the host structure subjected to (**a**) impact hammer (IH) vibrations (clamped) and (**b**) shaker (SV) vibrations (clamped); (**c**) impact hammer (IH) vibrations (adhesively bonded) and (**d**) shaker (SV) vibrations (adhesively bonded); (**e**) peak voltage ratio (V_TSAH-DISC_/V_DB-DISC_) for all four conditions.

**Figure 12:**
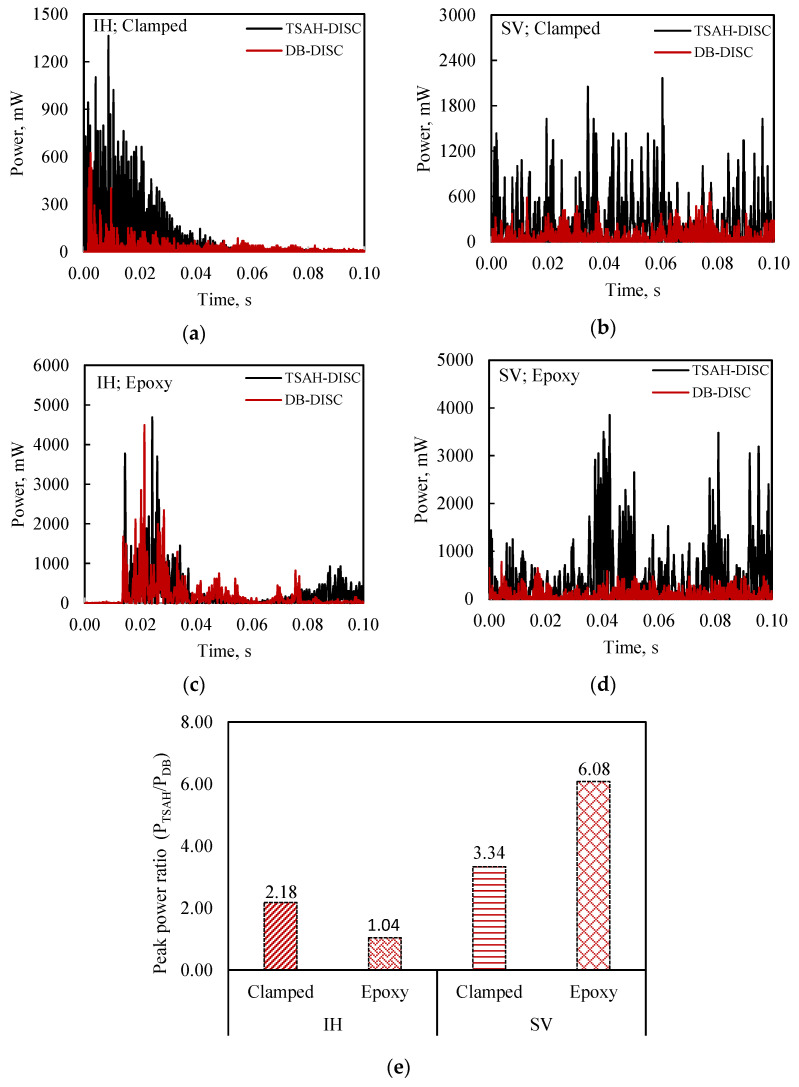
Comparison of power generated between TSAH-DISC and DB-DISC, with the host structure subjected to (**a**) impact hammer (IH) vibrations (clamped) and (**b**) shaker (SV) vibrations (clamped); (**c**) impact hammer (IH) vibrations (adhesively bonded) and (**d**) shaker (SV) vibrations (adhesively bonded); (**e**) peak power ratio (P_TSAH-DISC_/P_DB-DISC_) for all four conditions.

Thereafter, the G-clamps were removed and the TSAH-DISC was directly bonded to the host structure using epoxy adhesive for testing under IH and SV excitations. As in the case of MFC, a comparative analysis was made to determine the efficacy of the TSAH-DISC over the directly bonded disc PZT patch, by comparing their open circuit voltages and peak powers under IH and SV vibrations. The bonding condition and sequence of the experiments were followed as per the flow chart (see Figure 4). The open circuit voltage and power generated by TSAH-DISC and DB-DISC are shown in Figure 11 and Figure 12, respectively.

The voltage response of the TSAH-DISC and the DB-DISC under IH and SV excitations in the clamped condition is shown in Figure 11a and Figure 11b, respectively. Figure 11c and Figure 11d depict the voltage response when the TSAH-DISC was adhesively bonded to the host structure and of the DB-DISC, under IH and SV excitations, respectively.

From the plots, it can be observed that the open circuit voltage in the case of the TSAH-DISC is 1.3 to 4 times greater than that of the DB-DISC. The peak voltage ratio (V_TSAH-DISC_/V_DB-DISC_) for the above-mentioned cases is summarized in Figure 11e. It can also be observed that the peak voltage ratio in the case of the bonded TSAH-DISC is slightly higher when compared to that of the clamped condition for both IH vibrations, as in the case of the MFC patches (see Figure 9e). The power generated was determined using Equations (1) and (2) with the power measuring circuit. The power generated by the TSAH-DISC and DB-DISC when the host structure was subjected to IH and SV excitations under clamped conditions is shown in Figure 12a and Figure 12b, respectively. Further, the power generated when the TSAH-DISC was bonded to the host structure is shown in Figure 12c,d. In all cases, it can be observed that the power in the case of the TSAH-DISC is substantially high when compared to the DB-DISC. The peak power ratio (P_TSAH-DISC_/P_DB-DISC_) for the above-mentioned cases is summarized in Figure 12e, from where it can also be seen that the peak power ratio for all the cases is greater than one as in the case of MFCs.

Further, the peak power density of MFC and disc PZT patches was determined by dividing the absolute peak power value by the surface area of the PZT patch. The peak power density of the TSAH-MFC and DB-MFC was calculated as 7.61 mW/mm^2^ and 3.08 mW/mm^2^, whereas the peak power density of the TSAH-DISC and DB-DISC was calculated as 2.07 mW/mm^2^ and 0.34 mW/mm^2^, for SV vibrations when the TSAH was adhesively bonded to the host structure. It is evident from the power density values that the TSAH configuration was able to increase voltage and, consequently, power in both MFC and disc PZT patches. MFC patches, however, performed better than circular disc PZTs from power scavenging considerations.

### 5.4. Interpretation of Power Harvested from TSAH and DB Harvesters Using Peak Power Ratio and Average Power Ratio

A comparison between the peak and average power ratios is used to evaluate the power generated by both the harvesters. The degree of amplification that TSAH provides over DB is indicated by the peak power ratio (P_TSAH_/P_DB_). The average power ratio (Avg. P_TSAH_/Avg. P_DB_) indicates an estimate of the actual energy that can be stored in the battery or capacitor during harvesting (Avg. P_TSAH_/Avg. P_DB_). By using Equation (2), the total energy (E) produced by the harvester is determined by arriving at the area under the curve for the power (P) vs. time (*t*) plot as

(3) $$E=\int_{0}^{t}i^{2}(R_{1}+R_{2})dt$$

The average power (*P*_avg_) generated by the harvester is determined using Equation (3) and is defined as

(4) $$P_{avg}=\frac{E}{t}$$

Further, the average power ratio (Avg. P_TSAH_/Avg. P_DB_) is obtained by dividing the average power of the TSAH harvester by that of the DB harvester. The average power ratio is evaluated using Equation (4) when the host structure was subjected to shaker vibrations.

**Figure 13:**
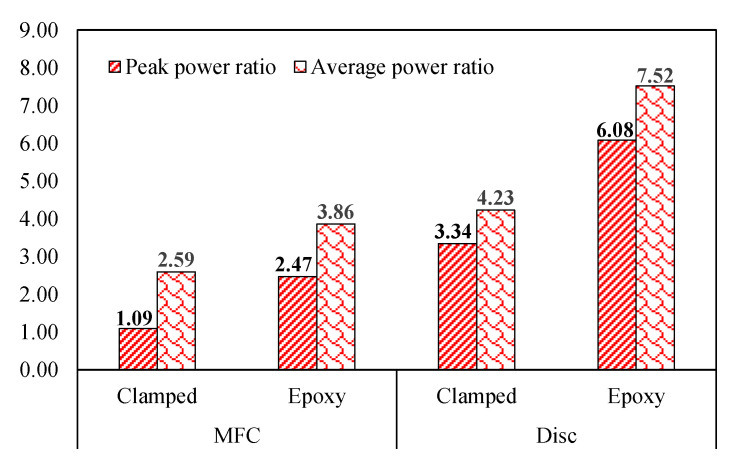
Comparison of peak and average power ratios.

Figure 13 illustrates the values of the peak and average power ratio for TSAH and DB harvesters under shaker vibrations. It can be observed from Figure 13 that the average power values are higher than the peak power values, which is justified as the power accumulated over time is greater than the power at any given instance. Though the power generated in the epoxy-bonded case is more in comparison to the clamped case, due to better connectivity, it is noteworthy to mention that the ratio of 1.09 to 7.52 is found to be more than one in both cases. It shows that a clamped connection can be an effective alternative considering the reusability of the TSAH. However, further investigations on the applicability of temporary connections are in progress.

## 6. Field Investigations

**Figure 14:**
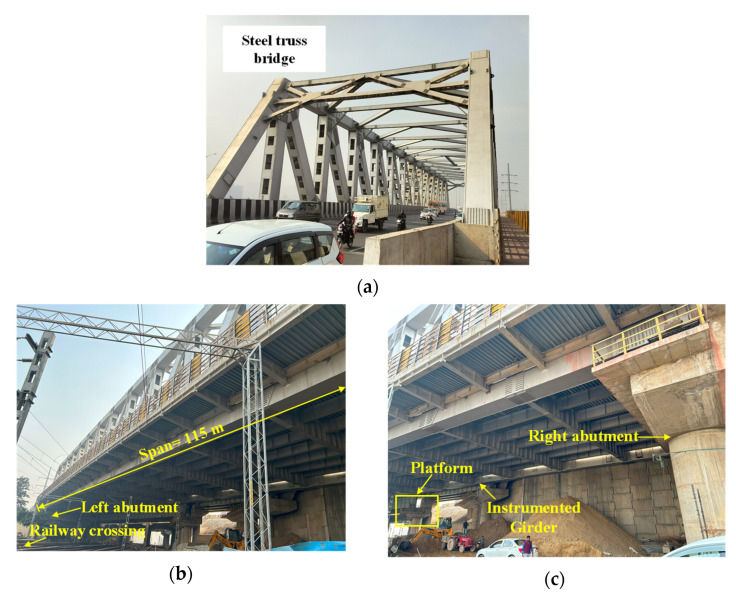
(**a**) Overall view of the bridge, (**b**) underside view of the bridge, and (**c**) location of instrumented girder.

This section presents the transition from lab benchmark studies to field conditions. It aims to validate the performance of the TSAH for traffic-induced vibrations of a bridge structure. The field experiments were carried out on Chipiyana bridge as shown in Figure 14, which is a road over-bridge (ROB) across a railway crossing, located in Ghaziabad on the Delhi–Meerut expressway, India.

The ROB is a truss-type steel structure with a skew in plan with an angle of around 35°. The ROB steel structure weighs 2385 metric tonnes and is Asia’s heaviest steel bridge, with a single span length of 115 m and a carriageway width of 21 m (Figure 14). As per the estimates of the National Highways Authority of India (NHAI), the mentioned ROB has a traffic volume of around 500,000 passenger car units per day.

**Figure 15:**
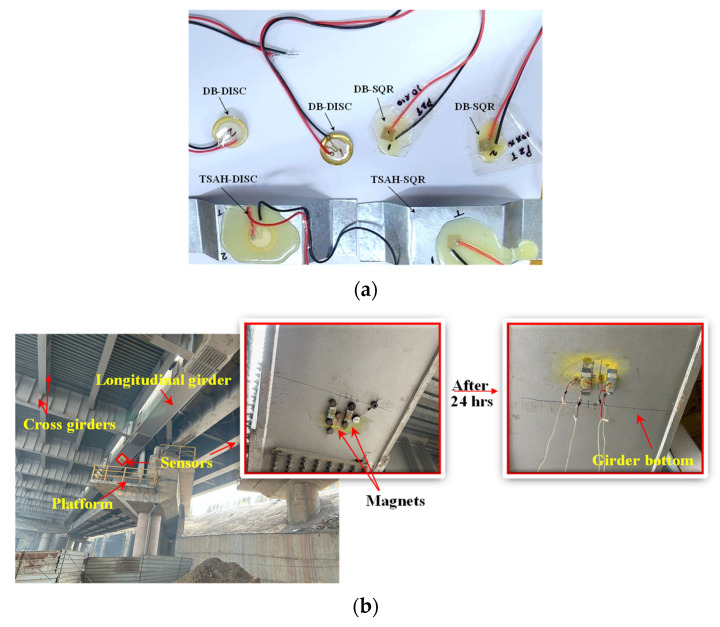
(**a**) Sensors before installation, (**b**) sensors bonded with epoxy adhesive were kept intact initially using magnets and were removed after bonding.

For field deployment, the soldered patches were made weather-resistant by coating them sufficiently with epoxy adhesive to protect them from the harsh environment in the field. One each of the TSAH-DISC and TSAH-SQR were fabricated at the laboratory, as shown in Figure 15a for field application. The bottom surface of the main girder of the ROB was made smooth by rubbing it with sandpaper, then the marking was conducted to ensure that all of them lay in one single line. Further, all the sensors were bonded to the bottom face of the longitudinal main girder of the ROB steel bridge using epoxy adhesive. To prevent the sensors from falling off the girder due to self-weight and vibrations caused by traffic, large magnets were used to hold them in place for 24 h, as shown in Figure 15b.

**Figure 16:**
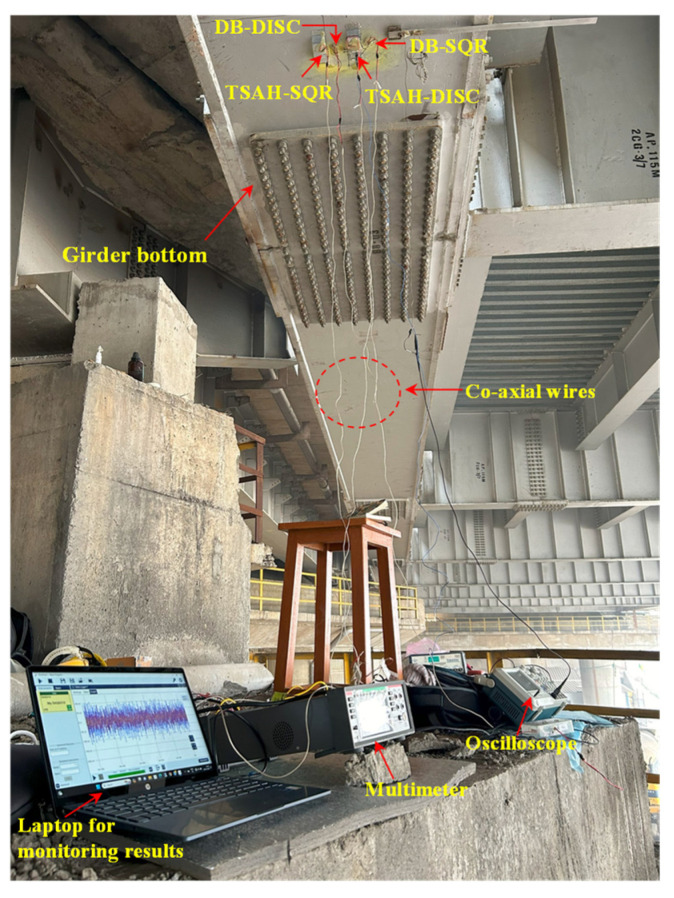
Experimental setup to acquire signals from the sensors from traffic-induced bridge vibrations.

**Figure 17:**
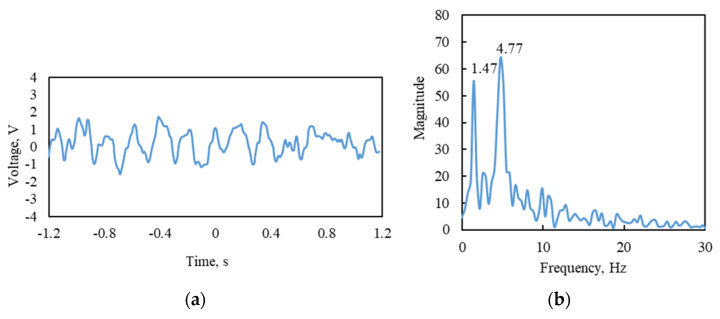
(**a**) Time domain of TSAH during vehicular movement and (**b**) FFT depicting the natural frequency of the bridge structure.

After ascertaining the proper bonding of the sensors to the bottom of the girder, the voltage and power measuring experiments were carried out under traffic to acquire the required data. The details of the experimental setup are shown in Figure 16. Time domain signals of the TSAH-PZT were acquired when the bridge was vibrating under traffic excitations, as shown in Figure 17a. The frequency domain of the voltage values that were transformed using the Fast Fourier Transform (FFT) function is shown in Figure 17b.

**Figure 18:**
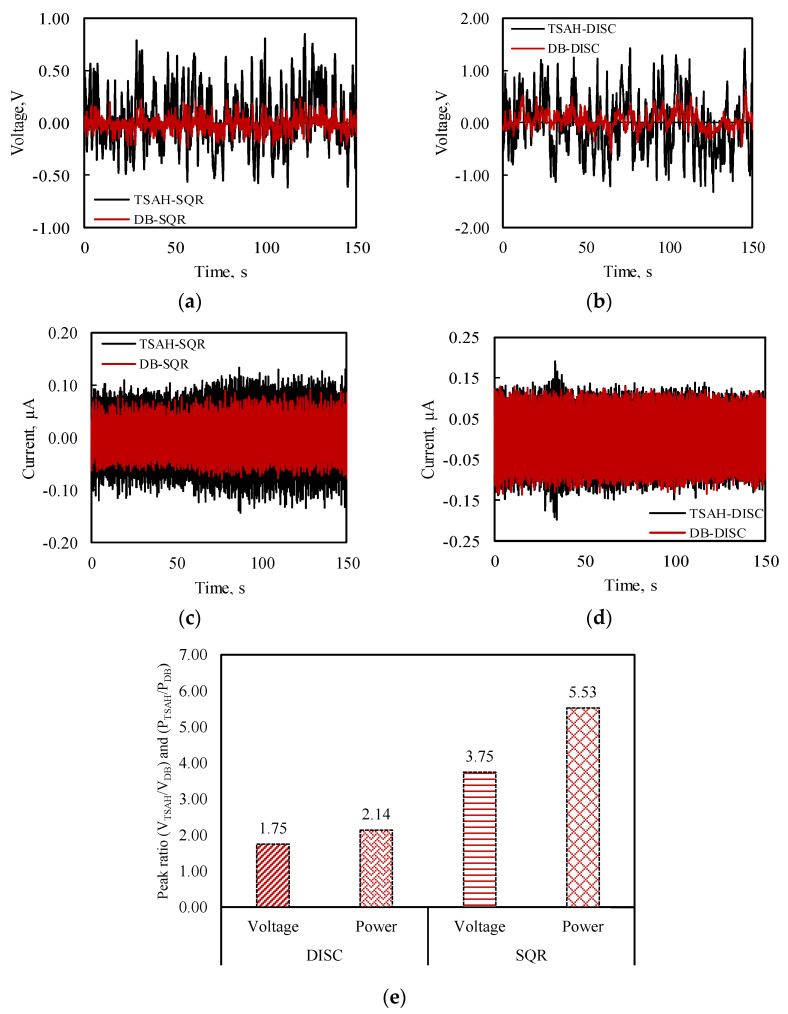
Comparison of (**a**) open circuit voltage generated with time (TSAH-SQR vs. DB-SQR), (**b**) open circuit voltage generated with time (TSAH-DISC vs. DB-DISC), (**c**) current generated with time (TSAH-SQR vs. DB-SQR), (**d**) current generated with time (TSAH-SQR vs. DB-SQR) and (**e**) peak ratios of voltage and power in case of square and disc PZTs for traffic-induced vibrations.

This test validated the proper working of the PZT-based sensors. The first natural frequencies of steel-type bridge decks for various spans and configurations, calculated as per the equations given by [63], were in the range of 0.70 to 2.72. Since the first peak frequency, i.e., 1.47 Hz (Figure 17a) lies in the above-calculated range, it was considered the first natural frequency of the bridge structure. Further, it was observed that the second peak is around four times the first frequency; hence, 4.77 Hz is likely to be the second natural frequency of the bridge structure. The open circuit voltage and the current generated by the sensors when the bridge was subjected to traffic-induced excitations were obtained with the help of a DAQ6510 Multimeter [64]. Initially, the open circuit voltage generated by all four types of harvesters cum sensors was acquired by connecting the sensors to the voltage channels of the multimeter. The acquired voltage signals were plotted when the bridge was subjected to vehicle-induced bridge vibrations. Figure 18a and Figure 18b show the voltage comparison of the TSAH-SQR vs. the DB-SQR and the TSAH-DISC vs. the DB-DISC, respectively. Further, the current generated was acquired by connecting the harvesters cum sensors to the power channels of the multimeter. Figure 18c and Figure 18d represent the current acquired by the TSAH-SQR vs. DB-SQR and TSAH-DISC vs. DB-DISC, respectively. The power ratio generated by the harvesters cum sensors was calculated from the measured current readings using Equation (5).

(5) $$P_{\mathrm{Ratio}\;}=\frac{{I^{2}}_{TSAH}}{{I^{2}}_{DB}}$$

The determined peak power ratio and voltage ratio for both the harvesters cum sensors are shown in Figure 18e. It is observed from Figure 18 that the voltage and power generated by the TSAH are much higher than that of the directly bonded sensors in the case of square and disc PZT patches. The peak voltage ratio (V_TSAH_/V_DB_) in the case of circular disc PZTs was 1.75, whereas, in the case of square PZTs, it was found to be 3.75 when the bridge was subjected to vehicular movements. The peak power ratio (P_TSAH_/P_DB_) in the case of circular disc PZTs was 2.14, whereas, in the case of square PZTs, it was 5.53 from the vehicle-induced vibrations. This establishes the efficacy of the TSAH over the DB harvesters in real-life field conditions as well.

## 7. Results and Discussions

The trapezoidal strain-amplifying configuration established from the numerical simulation was validated by conducting laboratory experiments as well as field investigations on a real-life bridge. The experiments were conducted to arrive at the voltage and power generated from the TSAH mounted on the host structure subjected to shaker vibration/impact type excitations.

- From the laboratory investigations, it is observed that in the case of MFC, the peak voltage ratio (V_TSAH-MFC_/V_DB-MFC_) ranges between 1.77 and 2.53, for different cases considered in this study.
- When the TSAH-MFC was clamped, the peak power ratio was 1.09 for SV vibrations, whereas when it was adhesively bonded, the peak power ratio increased to 2.47, which shows the better transfer of the strains in the latter case.
- Further, when the TSAH-MFC was clamped and subjected to IH vibrations, the peak power ratio was 6.73 as compared to 3.21 when it was adhesively bonded. This may be attributed to additional fluttering of the TSAH on sudden hits due to the impact type of excitations.
- The observations in the case of the disc PZT patch on the open circuit voltage are similar to those of MFC. The peak voltage ratio (V_TSAH-MFC_/V_DB-MFC_) ranges between 1.45 and 3.75 for different cases considered in this study. This ascertains the voltage amplification irrespective of the connection criteria and the type of vibration.
- In the case of the disc PZT patches, the peak power ratios for clamped and adhesively bonded scenarios were 3.34 and 6.08 for SV vibrations, respectively. When the TSAH-DISC was subjected to IH vibrations, the peak power ratios were obtained as 2.18 and 1.22 for clamped and adhesively bonded scenarios, respectively. The trend of strain amplification in the case of disc PZT patches is identical to that of MFC patches in all cases.
- Further, the average power ratios were evaluated under shaker vibrations for both TSAH and DB harvesters for different connection criteria. It was observed that the average power values are higher than the peak power ratios and are greater than one, which indicates that TSAH is an effective strain-amplifying energy harvester.

Since the proposed TSAH results in a manyfold amplification of the strain, this can be used as a potential energy harvesting device to drive piezo sensors.

For the case of the real-life structure, Chipiyana ROB, the following was observed:

- The peak voltage ratio (*V*_TSAH_/*V*_DB_) in the case of circular disc PZTs was 1.75, whereas, in the case of square PZTs, it was found to be 3.75. The lower amplification in the case of the field test can be attributed to the overly large beam depth in the case of the ROB, resulting in a relatively smaller incremental gain in terms of distance from the neutral axis.
- The peak power ratio in the case of square and disc PZT patches was obtained as 5.53 and 2.14, respectively.

It should be noted that the peak voltage and power ratios obtained are specific to the type of sensors and the TSAH that were considered in this study. Modification of any of the parameters may lead to a change in those values, but the amplification trend will remain similar. However, there are certain limitations to the TSAH, such as being prone to vandalism and deterioration over a long period due to the ambient weather conditions. Also, being made from metal, the TSAH may deform under heavy impacts during operation.

## 8. Conclusions and Future Recommendations

This study aimed to evaluate and establish the efficacy of the novel trapezoidal strain-amplifying configuration of PZT-based energy harvesters over the directly bonded harvesters in lab and field conditions. This proposed configuration is preferred in comparison to the curved configuration as it is suitable for brittle sensors and can be employed on steel constructions, as they are primarily externally attached. The TSAH can be replaced if the sensors fail to operate properly. It also allows for replacement and reuse, which contributes to the goal of sustainability. The results of the studies presented in the article conclude that in both field and laboratory investigations, the voltage and power generated by the TSAH were manyfold higher than those of the directly bonded harvesters cum sensors, thus, establishing the efficacy of TSAH harvesters cum sensors over directly bonded harvesters cum sensors.

The numerical investigations presented the preliminary study for the proof-of-concept. It was found that in comparison to thickness, the angle of inclination has a greater influence. Further, the research was extended to laboratory validation and field application, with the following conclusions:

(1) The peak voltage ratio of TSAH-MFC for SV vibrations is 2.53, which was observed to be higher than that generated by the curved energy harvester proposed in the earlier study by Krishnanunni et al. (2023) Ref. [54]. Hence, the TSAH serves as an effective energy harvester cum sensor to be used in steel construction.
(2) Both MFC and disc PZT patches were able to have their voltage and power amplified by the TSAH configuration, with the MFC patches performing better than the disc PZT patches.
(3) The voltage and power amplification were consistently observed regardless of the vibration type and connection requirements.
(4) The amplification factors varied in the range of 1.45 to 3.75 for peak voltage and 1.09 to 6.08 for peak power, depending on the type of vibration the host structure was subjected to and the connection criteria between the host and the secondary structures.
(5) The voltage ratio in the case of the SV vibrations was higher when compared to that of the IH vibrations, both in the case of MFCs and disc PZTs.
(6) Furthermore, this study suggests that clamping can be a suitable substitute for permanent bonding. Although the amplification in the clamped situation is less than in the adhesively bound connection, clamping enables the reusability of the TSAH.
(7) The TSAH was successful in amplifying the strains and, consequently, the voltage, even in real-life bridge structures subjected to traffic-induced vibrations.
(8) The average power ratios were higher than the peak power ratios in all the scenarios for SV vibrations, which indicates the potential ability of the TSAH for power storage in capacitors/batteries.

It is, however, observed that although the clamped configuration offers ease of installation and reusability, its performance shows a large spectrum of variation, possibly owing to the variation in clamping force. The variability in the case of clamped configuration is possibly due to the variation in the clamping force, which is most likely not consistent. The inconsistency in the case of clamped connection needs further investigation for standardization. Since this study focused on establishing the proof-of-concept for strain amplification of the TSAH, further studies shall be carried out to explore various connections, such as force-controlled clamping, welding/bolted connections, and connections using magnets.

## Figures and Tables

**Table 1:** Geometric configurations and material properties of the host and secondary structures.

Detail	Host Structure/Exterior Longitudinal Beam	Secondary Structure/Trapezoid Strain-Amplifying Plate (TSAP)
Longitudinal section	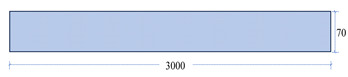	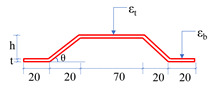
Cross-section	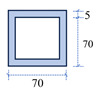	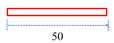
Material	Steel	Steel
Density, ρ (kg/m^3^)	7800	7800
Young’s modulus, E (GPa)	210	210

## Data Availability

Data shall be provided on a written request to the corresponding author.
